# Supplementary material for: A comprehensive map of alternative polyadenylation in African American and European American lung cancer patients
Source: Nat Commun. 2021 Sep 23;12:5605. doi: 10.1038/s41467-021-25763-5 (PMC8460807; doi:10.1038/s41467-021-25763-5)
Supplement: Supplementary file 1 — Supplementary Information [file 41467_2021_25763_MOESM1_ESM.pdf]

Supplementary information

A comprehensive map of alternative polyadenylation in African American and European American lung cancer patients

Zingone et al.

Supplementary Methods

ERCC dashboard analysis. The ERCC dashboard is a tool to assess the technical performance of a gene expression experiment. It generates standard metrics on the ERCC spike in transcript mixes. The ERCC spike in transcripts were designed to act as controls for microarray experiments (Baker et. al 2005). The polyA tail length was tailored for this and varies between 21 nt and 27 nt. The short polyA tail could bias the oligo dT priming, performed with QuantSeq. The overall transcript length distribution is rather short, compared to the annotated human transcripts. Each ERCC transcript is its own gene, lacking exon-exon junctions and transcript variants. For a QuantSeq experiment these variations are not considerable, since only the transcripts' 3' end is sequence. Aligned reads were processed with HTSeq-count (0.6.0) to obtain gene read counts using a combined GTF of GRCh.38.77 and the ERCC92-gtf provided by ThermoFisher. The single files were combined into a single matrix stripping the htseq-counts' special counters. The ERCC-dashboard (v. 1.4.0) (Munro et. al 2014) was used as described in results section

QC analysis of QuantSeq Rev data

Supplementary Fig.1a shows the normalized ERCC counts versus the spike amount [attomol nt/ µg total RNA] in log-log coordinates. In summary, the ERCC dashboard analysis [https:// doi.org/doi:10.18129/B9.bioc.erccdashboard] reviled no major flaws in the experiment's technical performance. The lowest detectable ERCC transcript is ERCC-00077 with a concentration of 3.66 attomoles/µl. The ERCC-dashboard computed a final concentration of 999.76 attomol nt/µg total RNA. The Pearson correlation of the dynamic range plot is 0.896. Supplementary Fig.1b shows the ROC plot of differential expression for three

defined ratios (range 0.9-0.991), indicating that the expected and observed data correlate well for ERCC transcript counts with absolute LFC greater 1. From the potential 69 ERCC transcripts 60 were detected. **c.** The limit of detection of ratio (LODR) is shown. The differential expression p-values are plotted against the average counts. To find the minimum signal required to detect differential expression confidentially, the read number is extracted, where the fitted curve's upper confidence interval is below the p-value threshold. In the case of the 4:1 ratio the minimum signal required is <1.1 reads, the model's minimal count. In the case of the 1:2 ratio set, the estimate is to have a base mean of 4.5 reads. Since the fitted curve of the 1:1.5 ratio is above the adjusted p-value threshold of 0.1, the LODR reports no minimal read count. The MA-plot depicts the ERCC spike in transcripts with the genomic background. **d.** The filled dots depict those results above the computed LODR.

Analysis of regulated APA events in lung cancer

While the adjusted p-value was used to define the significantly regulated sites in tumors versus normal, the fold change was then used to determine the direction of the change. Fold change > 0 indicated increased usage of the site analyzed and was defined as "enhanced", while fold change < 0 indicated decreased use of the site, and was labelled as "repressed". For genes with no significantly regulated sites, we selected two sites with the highest read counts across tumor and normal and classified them as "control up" and "control down" depending on their fold change.

If more than two sites were identified in one gene, only the two sites most significantly changed (adjusted p-value <0.05) were included for further analysis. If only one site had an adjusted p-value lower than 0.05, the second site with the highest read counts was selected. If no sites had

an adjusted $p<0.05$, then both sites were selected based on highest read counts. The PAS with adjusted value $p<0.05$ were labelled as significantly regulated sites.

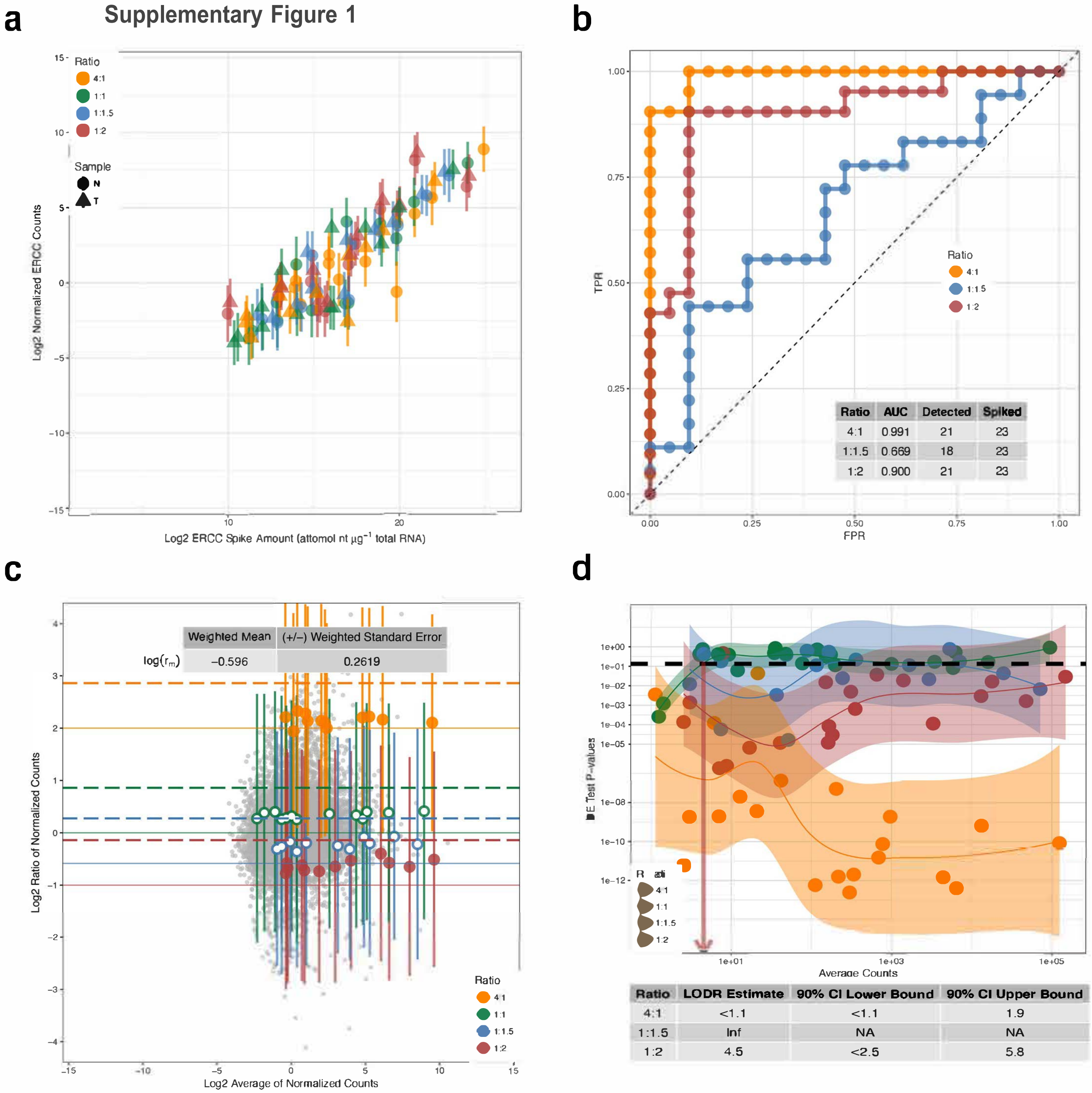

Supplementary Fig.1. ERCC **dashboard analysis. a** Normalized ERCC counts versus the spike amount [attomol nt / µg total RNA] in log-log coordinates. The lowest detectable ERCC transcript is ERCC-00077 with a concentration of 3.66 attomoles/ µl. The Erccdashboard computed a final concentration of 999.76 attomol nt / µg total RNA. The Pearson correlation of the dynamic range plot is 0.896. **b** shows the ROC plot of differential expression for the three sets with defined ratios. For the genes of the sets 1:2 and 4:1 an UC of 0.9 and 0.991 was yielded. It shows that the model is good explained by the observed ERCC transcript counts for absolute LFC greater 1. From the potential 69 ERCC transcripts 60 were detected. **c** The limit of detection of ratio (LODR) is shown. The differential expression test's P-values are plotted against the average counts. In order to find the minimum signal to detect differential expression confidentially, the read number is extracted, where the fitted curve's upper confidence interval is below the p-value threshold. In the case of the 4:1 ratio set it is <1.1 read, the models minimal count. In the case of the 1:2 ratio set, the estimate is to have a base mean of 4.5 reads. Since the fitted curve of the 1:1.5 ratio set is above the adjusted P-value threshold of 0.1, the LODR reports no minimal read count. **d** Modified MA-plot is shown. It shows the ERCC spike in transcripts with the genomic background. The filled dots depict those results above the computed LODR. Source data are provided as a Source Data file.

Supplementary Figure 2

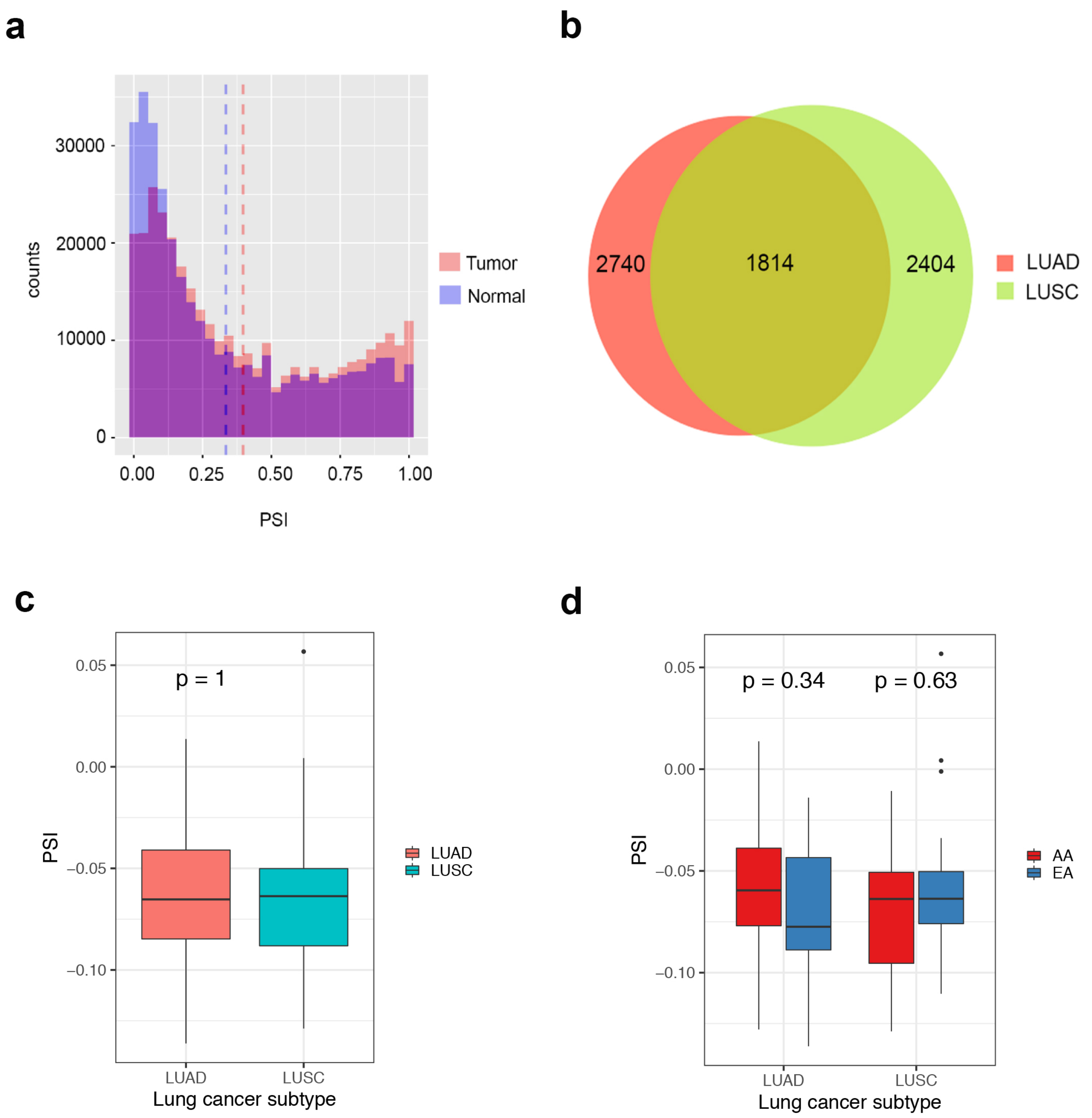

Supplementary Fig.2. Distribution of polyA index (PSI) in tumor and normal samples. **a** Higher PSI indicates shorter 3'UTR. **b** Shared and distinct APA events in tumor vs non-involved adjacent samples between lung adenocarcinoma (LUAD) and lung squamous cell carcinoma (LUSC). **c** The median 3'UTR length (tumor - normal) between LUAD and LUSC tumor samples. **d** The median 3' UTR (tumor - normal) in African Americans (AA) and European Americans (EA) for both LUAD or LUSC samples separately.

Supplementary Figure 3

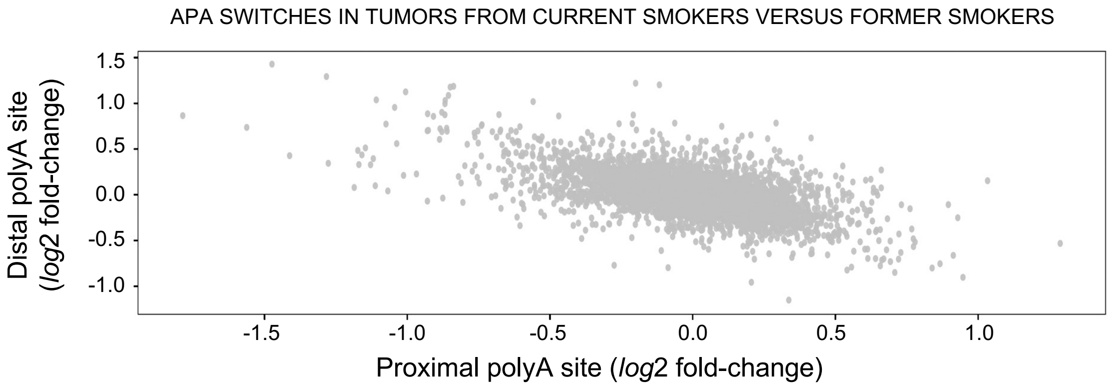

Supplementary Fig. 3. Comparison of APA events between current and former smokers. Grey dots indicate that there are no significant differences.

Supplementary Figure 4

a

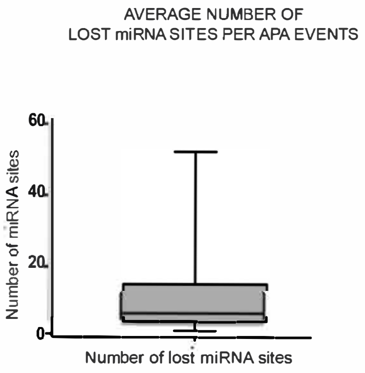

b

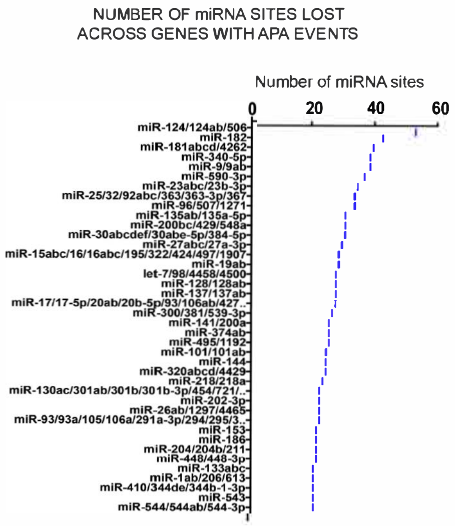

Supplementary Fig. 4. miRNA binding sites analysis across genes with APA events. **a** Average number of miRNA binding sites lost per miRNA transcript undergoing significant differential use of proximal versus distal polyA sites in lung cancer. **b** Summary of the numbers of binding sites lost per miRNA in mRNA transcripts undergoing significant differential use of proximal versus distal polyA sites in lung cancer.

Supplementary Figure 5

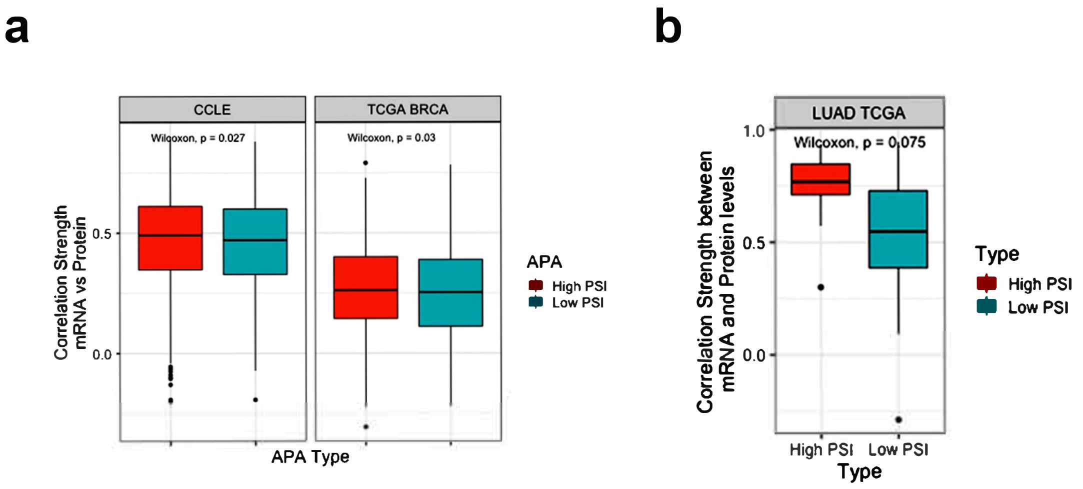

Supplementary Fig. 5. Relationship between 3'UTR length and mRNA protein correlation. **a** mRNA protein correlation among the top 10% and bottom 10% genes ranked by median polyA sites index (PSI) in CCLE samples and BRCA samples and **b** mRNA protein correlation in lung cancer samples.

Supplementary Figure 6

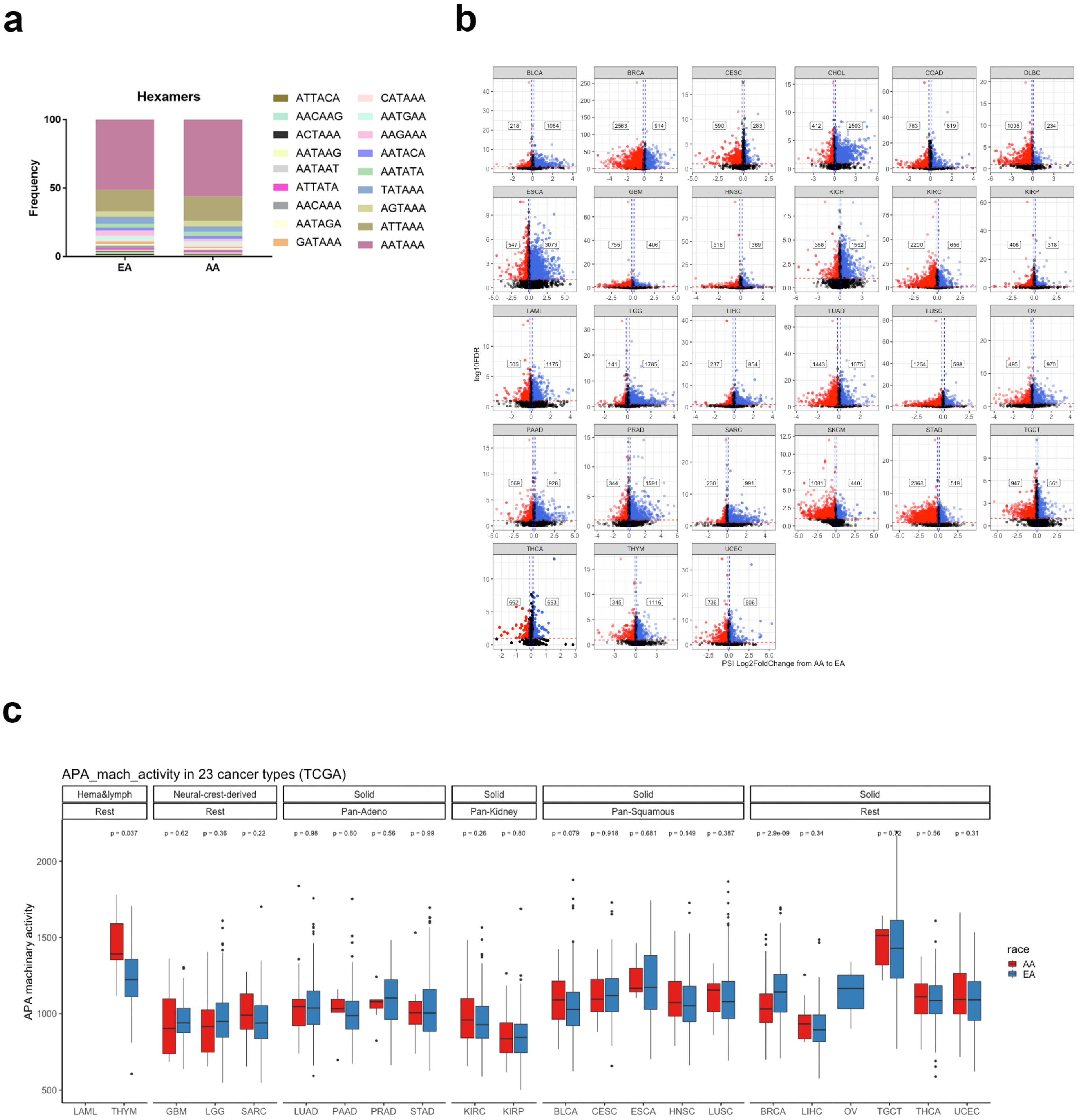

Supplementary Fig. 6. Population-specific use of poly(A) site (PAS) hexamers and APA events across cancer types in TCGA. a Use of PAS hexamers at regulated proximal sites in lung cancer in European Americans (EA) and African Americans (AA). **b** PA machinery genes score in EA (blue) and AA (red) across cancer types in TCGA.
