# Supplementary material for: A comprehensive map of alternative polyadenylation in African American and European American lung cancer patients
Source: Nat Commun. 2021 Sep 23;12:5605. doi: 10.1038/s41467-021-25763-5 (PMC8460807; doi:10.1038/s41467-021-25763-5)
Supplement: Supplementary file 3 — Description of Additional Supplementary Files [file 41467_2021_25763_MOESM3_ESM.pdf]

Description of Additional Supplementary Files

File Name: Supplementary Data 1
Description: **Distribution of genes with more than 1 polyadenylation site and detected in lung tissue.** List of genes with more than 1 polyA site detected in lung tissue and number of polyA sites present in each gene.

File Name: Supplementary Data 2
Description: **Classification of polyA pair sites among 3,531 genes with enhanced or repressed usage of proximal site.** Classification of regulated proximal and distal polyA pair sites of 3,531 genes based on their location in the gene. If both polyA sites are in the same exon are classified as same exon type. If the proximal site is part of a composite exon containing an internal 5' splicing site so that the two polyA sites are generated by alternative 5' splice site use, are classified as composite exon. If the proximal polyA site is in an exon that is fully skipped when the distal polyA site is used, they are classified as skipped exon.

File Name: Supplementary Data 3
Description: **Classification of polyA pair sites in genes with differentially regulated sites.** List of the number of regulated pairs sites based on their classification

File Name: Supplementary Data 4
Description: **Details of the regulated and non-significant APA events in lung cancer.** List of genes with more than 1 polyA site, their corresponding chromosome, their name and biotype, the nucleotide position of regulated proximal and distal sites in each gene, the region of the gene where the two sites are located, reads counts (column K and column O), and the analysis in the last few columns indicating the fold-change (proximal fc and distal fc) and p value in tumor versus non-involved tissue. Column P and column Q contain the sequence of the proximal and distal polyA site respectively. Colum X indicates the polyA pair type and in column Y genes are classified as enhanced or repressed depending on the proximal site usage if enhanced or repressed. Genes with no significant changes in the usage of sites are classified as control.

File Name: Supplementary Data 5
Description: **Polyadenylation site index (PSI) for each of the regulated APA sites in lung tumors.** Lists of 3,531 genes with regulated sites and their classification in repressed or enhanced. Each column contains the corresponding PSI index value for each tumor sample.

File Name: Supplementary Data 6
Description: **Histology specific changes in APA.** The file contains processed data in tumour versus non-involved tissues based on the histologic subtype of lung tumour samples (adenocarcinoma or squamous cell carcinoma).

File Name: Supplementary Data 7
Description: **Pathway analysis of mRNA transcripts with shortened and lengthened 3'UTRs.** Results of the Ingenuity Pathway Analysis with the list of all pathways significantly enriched associated with transcripts with shortened and lengthened 3'UTR separately. Fisher's exact test p-value< 0.05 was used. Results are expressed as -Log10(p-value). The list includes only pathways significant enriched with a -Log10(p-value) greater than 1.3.

File Name: Supplementary Data 8
Description: **Genes with significant APA events associated with survival.** List of genes whose PSI index is significantly associated with survival days across patients in a univariate test. Respective Hazard Ratio (column B) and significance (Column C) is provided. This is repeated via multivariate lasso regression and results are provided at the bottom. Two-sided log-rank test is used to compare the survival times between the high risk and low risk group. The pvalue of the log rank test statistic is commonly approximated by the chisquare distribution and thus only approximated in a higher limit.

File Name: Supplementary Data 9
Description: **Pathways enriched for genes with regulated APA associated with survival.** Gene ontology (GO) analysis of genes with regulated APA events associated with survival.

File Name: Supplementary Data 10
Description: **Number of miRNA binding sites lost in genes undergoing APA in lung cancer.** Summary of the number of miRNA binding sites lost per miRNA in genes undergoing to shortening 3'UTR by APA.

File Name: Supplementary Data 11
Description: **List of miRNA binding sites lost in genes undergoing APA in lung cancer.** List of genes and corresponding miRNA binding sites lost as results of APA.

File Name: Supplementary Data 12
Description: **Correlation between PSI and gene expression.** The correlation between PSI index and gene expression is provided for 3,531 genes with regulated sites. 1,376 genes have a significant correlation with expression. Respective Spearman Rho (column B) and significance (Column C) is provided. Two-tailed Wilcoxon rank sum test is used to compute the significance of the difference between medians.

File Name: Supplementary Data 13
Description: **Relationship between PSI and the strength of mRNA/protein correlations in CCLE and CTPAC (BRCA).** Relationship between PSI and the strength of mRNA/protein correlations in CCLE and CTPAC (BRCA). Respective gene ID (GeneID), correlation strength between expression and protein abundance (mRNAvsProt_Cor), whether the gene PSI is low or high (APA), HGNC gene name (GeneName) and the cohort where this correlation is computed (cohort, CCLE or BRCA TCGA).

File Name: Supplementary Data 14
Description: **Relationship between APA of non-coding microRNAs and expression. 14.1** List of 8 host miR genes undergoing to APA that correlates with expression in the same direction. Column D indicates shortening or lengthening of each miR as result of APA. 14.2 List of 10 host miR genes correlates with expression in the opposite direction. 14.3. List of isomiRs from the corresponding miRs listed in 14.1 that correlates with gene expression following the same trend. Column I indicate shortening or lengthening of isomiR as result of APA. 14.4. List of isomiRs from the corresponding miRs listed in 14.2 that correlates with gene expression with opposite trend. Column N indicate shortening or lengthening of isomiR as result of APA.

File Name: Supplementary Data 15
Description: **Relationship between APA of long non-coding RNAs and expression. 15.1.** List of long non-coding RNA genes associated with lung cancer survival, undergoing to APA in lung cancer and expression. 15.2. List of shortened and lengthened corresponding long non-coding RNA isoform genes and gene expression directly correlated with the length of their 3'UTR in the same direction. 15.3 List of shortened and lengthened corresponding long non-coding RNA isoforms with gene expression inversely correlated with the length of their 3'UTR in the opposite trend. 15.4 and 15.5 List of shortened and lengthened isoforms and isoforms expression directly and inversely correlated with their 3'UTR length in the same direction (column K) and in the opposite direction (column P) respectively

File Name: Supplementary Data 16
Description: **Details of the regulated and non-significant APA events in lung cancer among European Americans.** List of genes with more than 1 polyA site, their corresponding chromosome, their name and biotype, the number of polyA sites, the nucleotide position of regulated proximal and distal sites in each gene, the region of the gene where the two sites are located, reads counts (column P and column Q) and the analysis in the last few columns indicating the fold-change (proximal fc and distal fc) and p value in tumor versus non-involved tissues derived from European Americans patients. Colum X indicates the polyA pair type and in column Y genes are classified as enhanced or repressed depending on the proximal site usage if enhanced or repressed. Genes with no significant changes in the usage of sites are classified as control.

File Name: Supplementary Data 17
Description: **Details of the regulated and non-significant APA events in lung cancer among African Americans.** List of genes with more than 1 polyA site, their corresponding chromosome, biotype, and nucleotide position of the regulated proximal and distal polyA pair. Columns I and L indicate the gene region where the proximal and distal polyA site are located. The last few columns show the results of analysis with fold-change value and p value in tumor versus noninvolved tissues derived from African Americans patients. Column T indicates the polyA pair type and in column U genes are classified as enhanced or repressed depending on the proximal site usage if enhanced or repressed. Genes with no significant changes in the usage of sites are classified as control

File Name: Supplementary Data 18
Description: **Association between survival and PSI index for over 7,000 genes across 11,265 TCGA samples from 28 cancer types.** Here, the strength of association between PSI index and survival for each gene is provided (log (HR, natural base)) and the corresponding value (p-value), for European American and African American patients separately. Two-sided Wilcoxon rank sum test is used to compute the significance of the difference between medians

File Name: Supplementary Data 19
Description: **Genome-side relationship between APA machinery activity and PSI index in 11,265 TCGA samples from 28 cancer types.** Here the strength of this correlation (APA_machinary_activity_vs_PSI cor) and he HGNC gene (geneName) and the cohort where this correlation is computed (cohort, CCLE or BRCA TCGA) are provided.

File Name: Supplementary Data 20
Description: **The association of PSI load with driver gene mutation status.** Here, given a gene (Column Gene), the PSI fold change in mutated vs wildtype (FC_PSI(Mutated/WildType), and the corresponding p-value before (FC p-value) and after FDR correction (fdr FC-pvalue) are provided. Two-tailed Wilcoxon rank sum test is used to compute the significance of the difference between medians.

File Name: Supplementary Software 1
Description: https://github.com/ruppinlab/apa_lung.git
The link provides the codes generated to analyze publicly available datasets used in this manuscript.
