# Supplementary material for: A comprehensive map of alternative polyadenylation in African American and European American lung cancer patients
Source: Nat Commun. 2021 Sep 23;12:5605. doi: 10.1038/s41467-021-25763-5 (PMC8460807; doi:10.1038/s41467-021-25763-5)
Supplement: Supplementary file 5 — Supplementary Software 1 [file 41467_2021_25763_MOESM5_ESM.pdf]

Supplementary Software

R scripts can be assessed at the GitHub link:

https://github.com/ruppinlab/apa_lung.git

APA analysis: https://github.com/grexor/apa

README

1. System requirements

All software dependencies and operating systems (including version numbers) NA
Versions the software has been tested on NA
Any required non-standard hardware N/A

2. Installation guide

Instructions NA, this is an open source web-based platform
Typical install time on a "normal" desktop computer

3. Demo

Instructions to run on data

expressRNA implements alternative polyAdenylation (APA) analysis from 3'-end short-read targeted sequence data and long-read Nanopore / PacBio data. In general the analysis is flexible to accommodate diverse sequence data. The following steps are crucial for APA analysis:
map / align the reads to the reference with STAR short read aligner
construct a polyA atlas / database by clustering alignment ends (or beginnings, depending on the protocol)
filter out alignments from A-rich genomic regions (to avoid internal priming events in the atlas construction step)
recover the signal by computing the "expression" of polyA sites by clustering
define comparative analysis (set of control experiments vs. set of test experiments)
run DEXSeq analysis on the polyA sites
select two sites per gene to classify polyA events (shortening, lenghtening of genes)
perform motif analysis around regulated sites with RNAmotifs2
Gene Ontology Enrichment Analysis on APA regulated genes

We map (align) the reads to the reference genome with STAR. To build the genome index, we include GTF annotation files:

```
STAR --runMode genomeGenerate --genomeDir output_folder --genomeFastaFiles genome.fasta --sjdbGTFfile genome.gtf
```

After we align raw reads to the reference genome (default STAR settings) allowing soft clipping of bases and only reporting unique/best alignments are inclusive of at least 20% of the read length:

```
--outFilterMultimapNmax 1 --outFilterMatchNminOverLread 0.2 --outFilterScoreMinOverLread 0.2
```

We then iterate over each alignment taking the first (5') nucleotide as a potential polyA cleavage site. We check the surrounding genomic sequence [-30..10] for A-rich regions. If this region contains either AAAAAAA or #A > 8, the alignment is filtered out. However, if we find a polyA signal (PAS) / hexamer in the region [-30..0] then the alignment is kept.

We then combine the remaining alignments and construct the library polyA database. Since cleavage and polyadenylation is not nucleotide exact, we sort all positional values (genome wide) from highest to lowest and perform a clustering of values in the range [-25..25]. We produce a table (bed graph) of the genomic loci and annotate polyA sites to genes with 5KB downstream extension (or up to max half-

distance to downstream gene).

To compute the polyA count table, we take each genomic loci in the library polyA database and count the number of alignments (5' nucleotide) from each experiment separately in the vicinity of the polyA site (-5..5 nt). Counting considers all alignments from each experiment in the library. Filtering is only applied in the steps of constructing the library polyA database to account for internal priming events.

Expected output will be as shown in Supplementary Data 4.
Expected run time for demo on a "normal" desktop computer: NA

Demo data
http://www.expressrna.org/index.html?action=analysis&analysis_id=paper_final_all&module=es&pair_type=same

4. Instructions for use
How to run the software on your data
(OPTIONAL) Reproduction instructions
We encourage you to include instructions for reproducing all the quantitative results in the manuscript
See above
