# Supplementary material for: A comprehensive map of alternative polyadenylation in African American and European American lung cancer patients
Source: Nat Commun. 2021 Sep 23;12:5605. doi: 10.1038/s41467-021-25763-5 (PMC8460807; doi:10.1038/s41467-021-25763-5)
Supplement: Supplementary file 6 — Reporting Summary [file 41467_2021_25763_MOESM6_ESM.pdf]

natureresearch

Corresponding author(s): Brid Ryan

Last updated by author(s): June 29, 2021

Reporting Summary

Nature Research wishes to improve the reproducibility of the work that we publish. This form provides structure for consistency and transparency in reporting. For further information on Nature Research policies, seeAuthors & Referees and theEditorial Policy Checklist .

Statistics

For all statistical analyses, confirm that the following items are present in the figure legend, table legend, main text, or Methods section.

n/a	Confirmed	
☐	☒	The exact sample size (*n*) for each experimental group/condition, given as a discrete number and unit of measurement
☐	☒	A statement on whether measurements were taken from distinct samples or whether the same sample was measured repeatedly
☐	☒	The statistical test(s) used AND whether they are one- or two-sided<br>*Only common tests should be described solely by name; describe more complex techniques in the Methods section.*
☐	☒	A description of all covariates tested
☐	☒	A description of any assumptions or corrections, such as tests of normality and adjustment for multiple comparisons
☐	☒	A full description of the statistical parameters including central tendency (e.g. means) or other basic estimates (e.g. regression coefficient) AND variation (e.g. standard deviation) or associated estimates of uncertainty (e.g. confidence intervals)
☐	☒	For null hypothesis testing, the test statistic (e.g. *F*, *t*, *r*) with confidence intervals, effect sizes, degrees of freedom and *P* value noted<br>*Give P values as exact values whenever suitable.*
☒	☐	For Bayesian analysis, information on the choice of priors and Markov chain Monte Carlo settings
☒	☐	For hierarchical and complex designs, identification of the appropriate level for tests and full reporting of outcomes
☐	☒	Estimates of effect sizes (e.g. Cohen's *d*, Pearson's *r*), indicating how they were calculated

Our web collection on statistics for biologists contains articles on many of the points above.

Software and code

Policy information about availability of computer code

Data collection	The data were analysed using expressRNA.org and the "apa" module.
Data analysis	DEXseq was used for inference of differential exon use in the 3'UTR RNAseq data. http://bioconductor.org/packages/release/bioc/html/DEXSeq.html.<br>apa is a Python framework for processing and analysing 3'-end targeted sequence data to study alternative polyadenylation. apa interconnects pybio (basic handling of annotated genomes), RNAmotifs2 (analysis of regulatory motif clusters) and other open-source software (DEXSeq, STAR short-read aligner).<br><br>The inclusive nature of the framework, together with novel integrative solutions (differential polyA site usage and RNA-protein binding via RNA-maps, cluster motif analysis), results in the following computational capabilities: management of diverse high-throughput sequencing datasets (pre-processing, alignment, annotation), polyA site database (atlas) construction and comparison to existing polyA resources, identification of genes that undergo alternative polyadenylation (DEXSeq), identification of motifs influencing polyA site choice (RNAmotifs2), identification of motifs influencing alternative splicing (DEXSeq and RNAmotifs2), integration with iCLIP (RNA-protein binding) and computing RNA-maps.

For manuscripts utilizing custom algorithms or software that are central to the research but not yet described in published literature, software must be made available to editors/reviewers. We strongly encourage code deposition in a community repository (e.g. GitHub). See the Nature Research guidelines for submitting code & software for further information.

Data

Policy information about availability of data

All manuscripts must include a data availability statement. This statement should provide the following information, where applicable:
- Accession codes, unique identifiers, or web links for publicly available datasets
- A list of figures that have associated raw data
- A description of any restrictions on data availability

Complete raw data of our cohort is available as per request and has been deposited in compliance with the NIH data sharing to GEO repository under accession number GSE174330.

Field-specific reporting

Please select the one below that is the best fit for your research. If you are not sure, read the appropriate sections before making your selection.

- [x] Life sciences
- [ ] Behavioural & social sciences
- [ ] Ecological, evolutionary & environmental sciences

For a reference copy of the document with all sections, see nature.com/documents/nr-reporting-summary-flat.pdf

Life sciences study design

All studies must disclose on these points even when the disclosure is negative.

Sample size	Our primary aim was to create a lung cancer cohort with racial representation with enough statistical power for at least a few hypothesis simultaneously (multiple groups comparison or regressions).<br><br>We included RNA spike in controls in each library to infer our power to detect specific fold changes in polyA site usage between tumor and normal samples.
Data exclusions	No data are excluded
Replication	TCGA were used where possible to confirm the reproducibility of key findings. There are no other specific 3'UTR sequencing datasets of cancer to our knowledge. To verify the annotation of our polyA inference we mapped the annotations to two prior databases.
Randomization	All samples were randomized for RNA extraction, library preparation and sequencing based on tumor and normal annotation, race/ethnicity.
Blinding	All samples were blinded at the time of RNA prep, library preparation and sequencing.

Reporting for specific materials, systems and methods

We require information from authors about some types of materials, experimental systems and methods used in many studies. Here, indicate whether each material, system or method listed is relevant to your study. If you are not sure if a list item applies to your research, read the appropriate section before selecting a response.

Materials & experimental systems

n/a	Involved in the study	
x		Antibodies
x		Eukaryotic cell lines
x		Palaeontology
x		Animals and other organisms
	x	Human research participants
x		Clinical data

Methods

n/a	Involved in the study	
x		ChIP-seq
x		Flow cytometry
x		MRI-based neuroimaging

Human research participants

Policy information about studies involving human research participants

Population characteristics	All samples were taken prior to systemic treatment at the time of surgery. AA (45%) had a mean age of 63.7, were 78% male, 7%, 33% and 54% never, former and current smokers, respectively, 54% LUAD, 30% LUSC 7% other, and 50%, 30%, 15% and 5% stage 1, 2, 3 and 4, respectively. EA (55%) had a mean age of 65.2, were 67% male, 6%, 37% and 56% never, former and current smokers, respectively, 38% LUAD, 38% LUSC 20% other, and 67%, 23%, 6% and 4% stage 1, 2, 3 and 4, respectively.
Recruitment	Patients are approached at the time of surgery and asked to consent to the study having been given detailed information regarding the purpose and background of the study. Trained interviewers administer a detailed questionnaire to each patient. We are not aware of any biases in patient recruitment.
Ethics oversight	The IRBs of the NCI and University of Maryland Medical System approved this study and it is registered on clinicaltrials.gov [https://clinicaltrials.gov/ct2/show/NCT00339859].
